# Elevated Homocysteine Levels Predict Hospital-Acquired Pneumonia and Poor Functional Outcomes in Primary Intracerebral Hemorrhage

**DOI:** 10.3389/fneur.2022.926963

**Published:** 2022-06-24

**Authors:** Jun Peng, Guanghua Zhu, Sheng Xiao, Shucheng Liu

**Affiliations:** ^1^Department of Neurology, The First Affiliated Hospital, Hengyang Medical School, University of South China, Hengyang, China; ^2^Department of Urology, The First Affiliated Hospital, Hengyang Medical School, University of South China, Hengyang, China

**Keywords:** homocysteine, primary intracerebral hemorrhage, hospital-acquired pneumonia (HAP), poor outcome, restricted cubic spline (RCS)

## Abstract

**Background:**

Homocysteine (Hcy) has been extensively acknowledged to be correlated with inflammation. In this study, the relationship between Hcy and hospital-acquired pneumonia (HAP) in primary intracerebral hemorrhage (pICH) was explored.

**Methods:**

We conducted a hospital-based study on screened eligible patients with primary intracerebral hemorrhage admitted within 24 h after symptom onset from January 2019 to June 2021. The associations between Hcy and HAP and poor outcomes in pICH were investigated using univariate and multivariate logistic regression analyses. The predictive accuracy of Hcy was assessed by the receiver operating characteristic curve and the optimal cutoff value of Hcy was determined by Youden Index. The patterns and magnitudes of associations between Hcy and HAP and poor outcomes were evaluated using a restricted cubic spline (RCS).

**Results:**

A total of 579 patients with pICH were included in the study. Hcy level was significantly higher in patients with HAP and poor outcomes (*p* < 0.001). The univariate and multivariate logistic regression analyses demonstrated that elevated Hcy was independently associated with both HAP and poor outcomes (*p* < 0.001). Furthermore, receiver operating characteristic analysis indicated that Hcy exhibited a moderate predictive accuracy for both HAP and poor outcomes after pICH. The RCS model showed that there were linear relationships between Hcy and HAP and poor outcomes.

**Conclusions:**

Higher Hcy level was independently associated with HAP and poor outcomes in patients with pICH.

## Introduction

Intracerebral hemorrhage (ICH) remains one of the most common critical diseases in the neurological field, accompanied by a poor prognosis due to the limited treatment options (1). Patients with ICH are generally at high risk of suffering hospital-acquired pneumonia (HAP) during hospitalization, which is reported markedly correlated with poor functional outcomes and mortality (2). Accordingly, it is important to identify risk factors of HAP to improve clinical outcomes. A variety of risk factors for HAP after ICH have been identified, namely, mechanical ventilation, tube feeding, dysphagia, older age, male sex, ICH severity, heart diseases, pulmonary diseases, intubation, dysphagia, current smoking, and lymphopenia (2–5). Generally, an appropriate predictor should be both easily acquired and effectively intervened through existing management to improve the outcomes. Nevertheless, most of the current markers failed to meet these criteria. Thus, we attempt to explore an appropriate biomarker for predicting HAP after ICH.

Homocysteine (Hcy) is a sulfur-containing amino acid produced by methionine metabolism and is mainly eliminated through the kidneys. Over the last decade, a strong association between Hcy and induction of inflammatory determinants in both human and experimental models has been demonstrated (6–9). A previous study has proved that a high admission Hcy level is independently associated with HAP in patients with acute ischemic stroke (10). Based on this, we proposed the hypothesis that Hcy was an independent predictor for HAP in primary ICH.

Herein, in the present study, we explored the correlation of elevated Hcy with HAP and 3-month functional outcomes in primary ICH. Furtherly, we attempted to determine the predictive accuracy and the cut-off values of Hcy for HAP and 3-month poor outcomes after ICH.

## Materials and Methods

### Study Design and Participants

We conducted a prospective cohort study. Consecutive patients admitted to our department within 24 h from the onset of ICH from January 2019 to June 2021 were investigated. The exclusion criteria included the following: (1) patients with ICH who were attributed to any secondary causes, namely, arteriovenous malformation, aneurysmal hemorrhage, brain tumor, brain trauma, hemorrhagic transformation of ischemic infarction, thrombocytopenia, and coagulation disorders; (2) incomplete baseline clinical data; (3) the patients with primary intraventricular hemorrhage; and (4) history of infectious diseases or diagnosed with pneumonia within 48 h from admission. This study was conducted according to the guidelines of the Declaration of Helsinki and approved by the Institutional Ethical Committee of First Affiliated Hospital of the University of South China.

### Clinical and Laboratory Data

Demographic variables included age, sex, hypertension, coronary heart disease, diabetes mellitus, hyperlipidemia, smoking, and alcohol consumption. Baseline clinical data obtained on admission included systolic blood pressure, diastolic blood pressure, and blood sugar. The admission National Institute of Health Stroke Scale score and Glasgow Coma Scale (GCS) score, respectively, referred to for evaluating the neurological deficit and level of consciousness were collected and recorded. Surgical interventions mainly included hematoma evacuation, external ventricular drainage, and decompressive craniectomy. Intubation at admission was recorded. Laboratory tests were performed on admission. Serum levels of white blood cells, C-reaction protein (CRP), interleukin 6, and procalcitonin were measured at admission using standard laboratory methods. Serum levels of Hcy were measured at admission. Then, the baseline characteristics of the patients were compared according to the quartile groups (Q1–Q4). For all measurements, levels that were not detectable were considered to have a value equal to the lower limit of detection of the assay. Determinations were performed in an independent laboratory blinded to clinical and neuroimaging data.

### Neuroimaging Analysis

The investigators reviewing the CT scanning were blinded to clinical information. Head CT scans were obtained at admission. On the initial CT, hematoma location and presence of intraventricular extension were documented. Hematoma volumes were measured using the ABC/2 method (11), and intraventricular bleeding was not included in the volume calculations.

### Outcomes

The primary endpoint was HAP. We referred to HAP as that which appeared as of 48 h from hospital admission, whether or not related to mechanical ventilation (12, 13). The typical symptoms of pneumonia included fever, cough, pleurisy, dyspnea, and increased sputum production. The radiographic appearance of pneumonia can be highly variable which included lobar consolidation, bilateral interstitial infiltrates, pleural effusion, lobe cavitary lesions, diffuse bronchiectasis, and pulmonary abscess. HAP was diagnosed on the combination of the above common symptoms with consistent radiographic findings that were mentioned above. The secondary outcome was the poor functional outcome at 3 months, which was defined as the modified Rankin Scale of 4–6. The 3-month functional status was evaluated by a researcher at an outpatient clinic or through a structured telephone interview, who was blinded to the patients' lab data and clinical data from the initial admission.

### Statistical Analysis

Continuous variables were displayed as means (SDs) or medians [interquartile ranges (IQRs)] using Student's *t*-test or Mann–Whitney *U*-test as appropriate. Categorical variables were compared using the χ^2^ test. We used univariate analysis for comparing the variables to identify the possible significant predictors for HAP and poor outcomes. Subsequently, a stepwise multiple logistic regression was performed by using the remaining predictors (candidates for independent variables based on the results of univariate regression analysis) to determine the best sets of predictors. Receiver operating characteristic (ROC) analyses were conducted to determine the predictive value of Hcy for HAP and poor outcomes after ICH. And the optimal cutoff value for Hcy was obtained by the Youden index. The pattern and magnitude of associations between Hcy and HAP were evaluated using a restricted cubic spline (RCS) with 5 knots (at 5th, 27.5th, 50th, 72.5th, and 95th). Finally, the mediation of the effect of Hcy on the poor outcome by HAP was tested using the R package mediation. Statistical analyses were performed using SPSS (ver.23.0; SPSS Inc., IL, USA) and R software, version 4.0.2 (The R Foundation for Statistical Computing, Vienna, Austria; http://www.R-project.org/). A two-sided *p* < 0.05 was considered statistically significant.

## Results

### Baseline Clinical Characteristics

**Figure 1:**
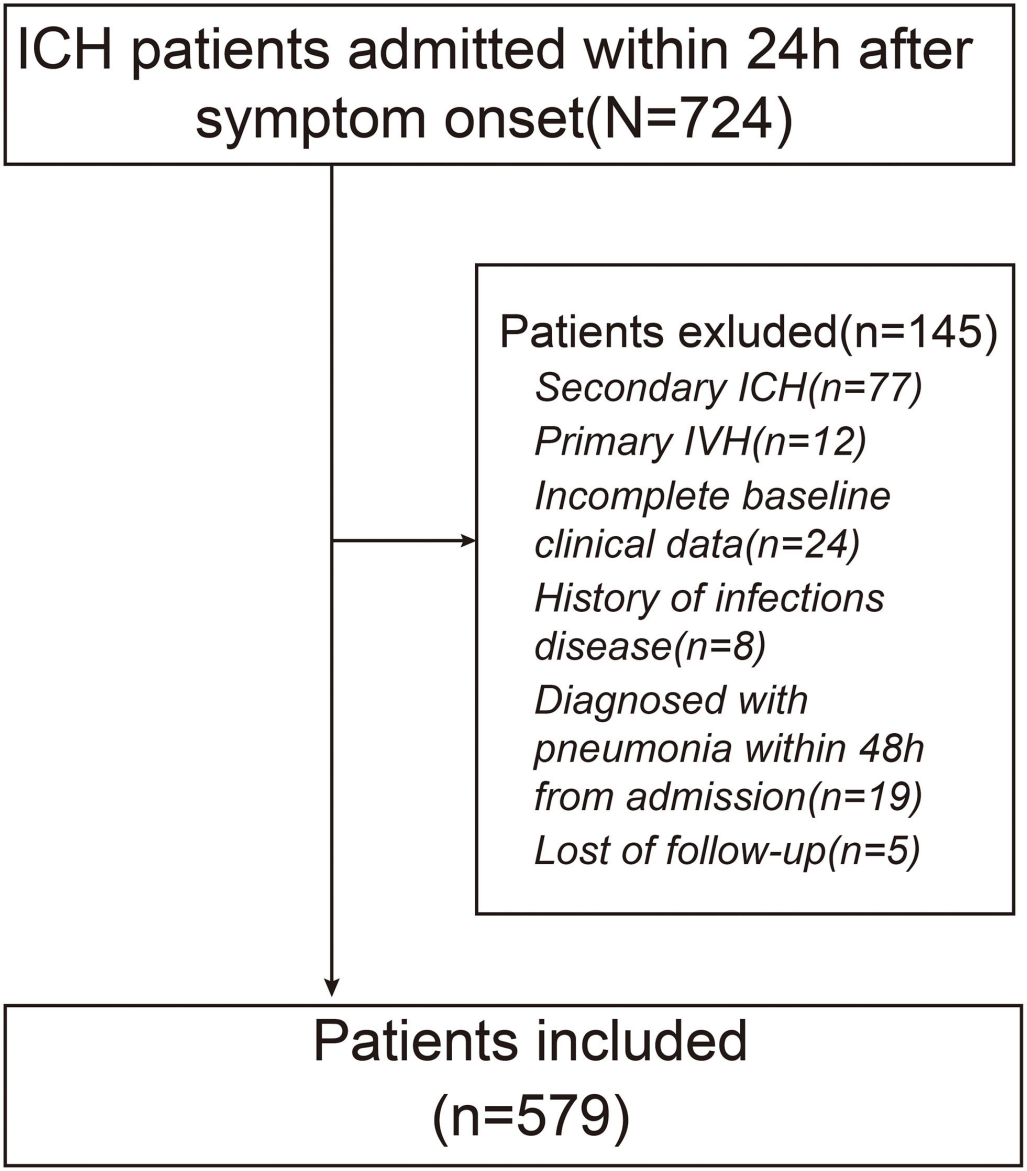
Flowchart of study patients.

A total of 579 patients were included in the final study. A flowchart of the study population is shown in Figure 1. Among the eligible participants, the mean age was 61.4(±11.5) years with predominantly men (69.8 vs. 30.2% women). The baseline GCS score was 9.74(±2.05). The baseline hematoma volume was 21.0 ml (IQR: 11.0–35.0 ml). Overall, the mean level of Hcy was 19.44(±8.10) μmol/l. The 579 subjects were divided into 4 groups according to the quartile value of the Hcy levels measured at baseline. There were significant differences in GCS scores, hematoma volume, and the proportion of patients undergoing surgery among different groups (*p* < 0.05). In addition, more patients with higher Hcy underwent HAP and 3-month poor outcome (*p* < 0.001). The clinical characteristics of the participants are summarized in Table 1.

**Table 1:** Baseline characteristics of participants according to Hcy quartiles.

**Characteristics**	**Hcy**					***p***
	**Overall**	**Q1:≤13.20** **(*n* = 147)**	**Q2:13.30–17.80** **(*n* = 144)**	**Q3:17.9–24.80** **(*n* = 144)**	**Q4:≥24.90** **(*n* = 144)**	
Age (years), M (SD)	61.43 (11.5)	60.4 (11.0)	61.5 (11.4)	60.4 (12.0)	63.4 (11.5)	0.094
Sex (female), *n* (%)	175 (30.2)	49 (33.3)	48 (33.3)	37 (25.7)	41 (28.5)	0.4
**Medical history**, ***n*** **(%)**
Hypertension	405 (69.9)	105 (71.4)	101 (70.1)	99 (68.8)	100 (69.4)	0.965
Diabetes mellitus	161 (27.8)	37 (25.2)	40 (27.8)	38 (26.4)	46 (31.9)	0.597
Coronary heart disease	107 (18.5)	29 (19.7)	28 (19.4)	21 (14.6)	29 (20.1)	0.582
Hyperlipidemia	191 (33.0)	46 (31.3)	40 (27.8)	47 (32.6)	58 (40.3)	0.143
Smoking	398 (68.7)	101 (68.7)	99 (68.8)	93 (64.6)	105 (72.9)	0.507
Alcoholism	283 (48.9)	72 (49.0)	65 (45.1)	77 (53.5)	69 (47.9)	0.557
**Clinical status on admission**
Blood sugar (mmol/L), median [IQR]	7.90 [6.10, 10.45]	8.10 [5.80,10.70]	8.15 [6.15, 10.62]	7.65 [6.10,10.03]	8.05 [6.40,10.60]	0.791
SBP (mmHg), M (SD)	179.2 (30.4)	174.4 (30.0)	181.5 (30.6)	181.4 (30.8)	179.5 (29.9)	0.151
DBP (mmHg), M (SD)	97.3 (16.6)	94.8 (15.1)	98.6 (17.6)	97.8 (16.7)	98.1 (16.7)	0.188
GCS score, M (SD)	9.74 (2.05)	9.81 (2.10)	9.96 (2.02)	10.01 (1.99)	9.17 (2.01)	0.001
NIHSS score, median [IQR]	10.0 [5.0, 17.0]	10.0 [5.0,16.0]	10.5 [6.0, 18.0]	9.0 [4.0, 15.0]	11.0 [6.0, 17.0]	0.29
**Radiographic status on admission**
Hematoma location, *n* (%)						0.878
Brain stem	33 (5.7)	7 (4.8)	10 (6.9)	5 (3.5)	11 (7.6)	
Cerebellum	25 (4.3)	7 (4.8)	5 (3.5)	6 (4.2)	7 (4.9)	
Basal ganglia	252 (43.5)	65 (44.2)	60 (41.7)	68 (47.2)	59 (41.0)	
Thalamus	149 (25.7)	41 (27.9)	33 (22.9)	36 (25.0)	39 (27.1)	
Lobe	120 (20.7)	27 (18.4)	36 (25.0)	29 (20.1)	28 (19.4)	
Hematoma volume (ml), median [IQR]	21.0 [11.0, 35.0]	19.0 [10.0,32.5]	18.0 [9.0,29.3]	19.5 [12.0, 34.5]	28.0 [14.0,44.0]	<0.001
IVE, *n* (%)	271 (46.8)	59 (40.1)	80 (55.6)	65 (45.1)	67 (46.5)	0.065
**Laboratory testing**
WBC (×10^9^/L), median [IQR]	7.7 [5.6, 10.7]	7.5 [5.7, 10.5]	7.7 [5.3, 10.6]	7.3 [5.5, 10.6]	7.9 [6.0, 11.0]	0.579
CRP (mg/L), median [IQR]	12.0 [7.0, 18.0]	12.0 [8.0, 18.0]	13.0 [7.0, 19.3]	10.0 [6.0, 17.3]	12.0 [6.0, 18.0]	0.081
PCT (ng/ml), median [IQR]	0.13 [0.07, 0.34]	0.14 [0.07,0.37]	0.16 [0.07, 0.37]	0.14 [0.07, 0.33]	0.10 [0.06, 0.25]	0.105
IL-6 (pg/ml), median [IQR]	29.0 [18.0, 43.0]	28.0 [18.0, 47.0]	29.0 [17.0, 40.3]	29.5 [18.8, 44.0]	29.5 [16.8, 44.0]	0.714
Intubation	99 (17.1)	23 (15.6)	23 (16.0)	29 (20.1)	24 (16.7)	0.728
Surgery	157 (27.1)	41 (27.9)	32 (22.2)	30 (20.8)	54 (37.5)	0.006
**Outcomes**
HAP, *n* (%)	144 (24.9)	16 (10.9)	10 (6.9)	45 (31.2)	73 (50.7)	<0.001
3-month poor outcome, *n* (%)	173 (29.9)	29 (19.7)	19 (13.2)	50 (34.7)	75 (52.1)	<0.001

*There were significant differences in GCS scores, hematoma volume, and the proportion of patients undergoing surgery among different groups (p < 0.05). More patients with higher Hcy underwent HAP and 3-month poor outcomes (p < 0.001)*.

### Association Between Hcy and HAP

**Figure 2:**
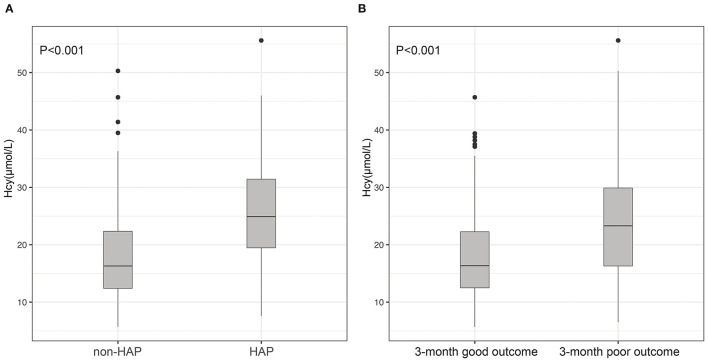
Differences of Hcy between patients with ICH and with and without HAP **(A)**; differences of Hcy between patients with ICH with 3-month good outcome and poor outcome **(B)**. **(A)** Compared with patients without HAP, levels of Hcy in those with HAP were significantly high (*p* < 0.001); **(B)** patients with ICH having poor outcomes had much higher Hcy compared with those with good outcomes (*p* < 0.001).

**Figure 3:**
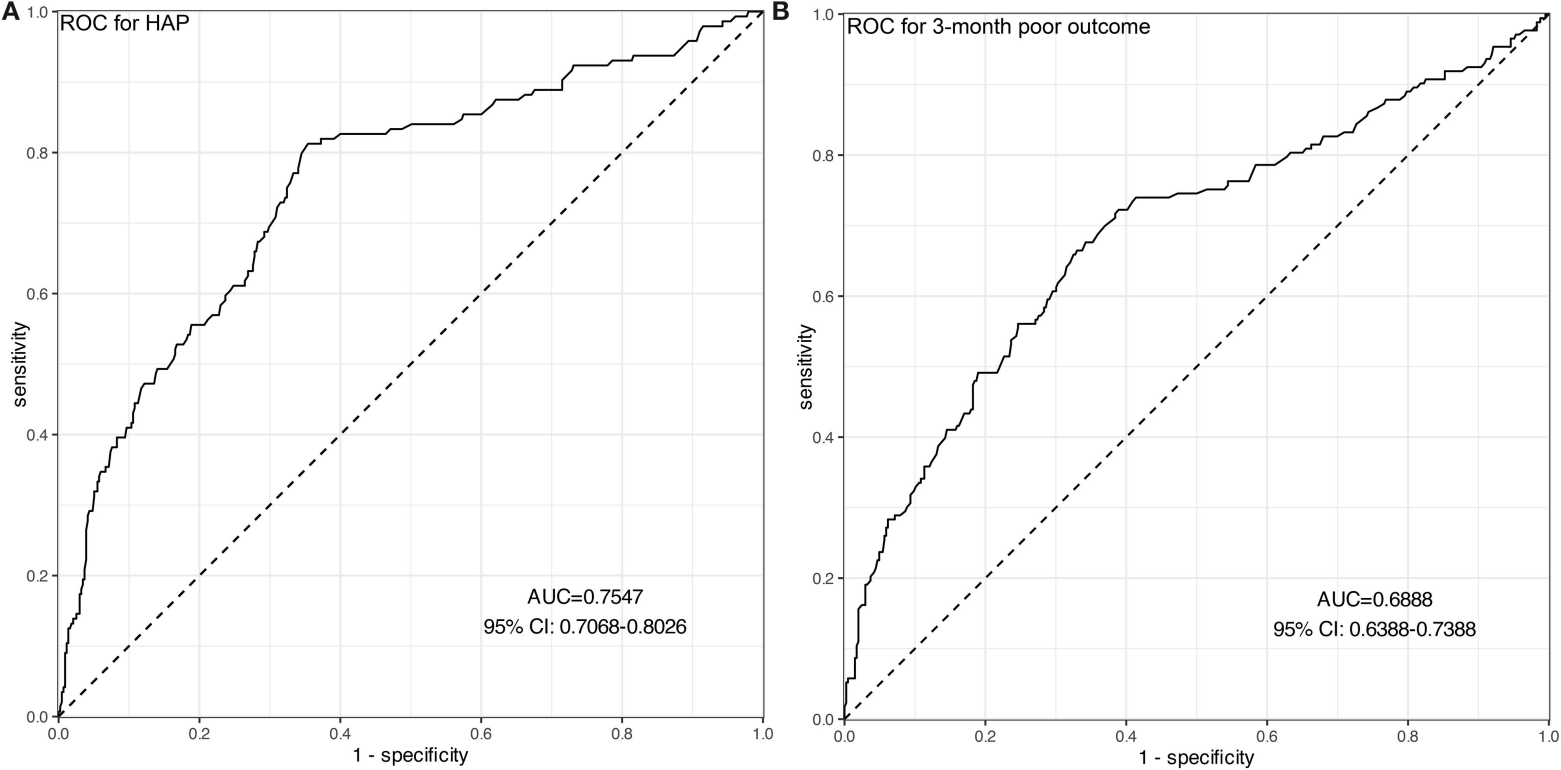
ROC curves of Hcy for predicting HAP **(A)** and 3-month poor outcomes **(B)**. **(A)** The AUC of Hcy was 0.755 with 95% CI: 0.707–0.803 (*p* < 0.001), indicating a moderate predictive ability for the development of HAP. **(B)** The AUC of Hcy was 0.689 with 95% CI: 0.639–0.739 (*p* < 0.001), indicating a moderate predictive ability for 3-month poor outcomes.

A total of 144 (24.9%) patients experienced HAP during their hospital stay. The occurrence of HAP was markedly increased accompanied by the increase of Hcy. Compared with patients without HAP, levels of Hcy in those with HAP were significantly high (*p* < 0.001; Figure 2A). Univariate analysis revealed significant differences in the history of diabetes mellitus (*p* = 0.011), age (*p* = 0.04), baseline GCS scores (*p* < 0.001), baseline hematoma volume (*p* < 0.001), intubation (*p* < 0.001), levels of CRP (*p* = 0.034), and Hcy (*p* < 0.001) for HAP prediction. Subsequently, the predictors (candidates for independent variables based on the results of univariate regression analysis: the history of diabetes mellitus, age, baseline GCS scores, baseline hematoma volume, intubation, and levels of CRP and Hcy) were included in a stepwise multiple logistic regression. The results showed that Hcy [odds ratio (OR): 1.122, 95% CI: 1.091–1.156, *p* < 0.001], intubation (OR: 3.479, 95% CI: 2.062–5.887, *p* < 0.001), and hematoma volume (OR: 1.031, 95% CI: 1.018–1.043, *p* < 0.001) were associated with HAP. Of note, there was still an independent association between Hcy and HAP after the factors of intubation and hematoma volume adjusted (Table 2). In ROC analysis, the area under the curve (AUC) of Hcy was 0.755 with 95% CI: 0.707–0.803 (*p* < 0.001), indicating a good association between homocysteine levels and the development of HAP (Figure 3A). The cut-off point for predicting HAP was 18.60 μmol/l with the sensitivity, specificity, positive predictive value (PPV), negative predictive value (NPV), and accuracy of 81.3, 64.6, 43.2, 91.2, and 68.7%, respectively (**Table 4**).

**Table 2:** Univariate and multivariate analyses of the potential predictors for HAP.

**Univariate selection**	**HAP**		
	**OR**	**95% CI**	***p***
Age	1.018	1.001–1.035	0.04
Sex	0.935	0.614–1.406	0.75
**Medical history**
Hypertension	1.012	0.674–1.538	0.954
Coronary heart disease	1.024	0.623–1.644	0.923
Hyperlipidemia	1.156	0.774–1.712	0.475
Diabetes mellitus	1.690	1.126–2.525	0.011
Smoking	1.091	0.728–1.656	0.676
Alcoholism	0.986	0.676–1.438	0.941
**Clinical status on admission**
Blood sugar	0.993	0.935–1.053	0.828
SBP	1.004	0.998–1.010	0.208
DBP	1.006	0.994–1.017	0.313
GCS score	0.849	0.773–0.930	<0.001
NIHSS score	0.999	0.974–1.023	0.919
**Radiographic status on admission**
**Hematoma location**
Cerebellum	0.726	0.213–2.328	0.595
Basal ganglia	0.672	0.309–1.554	0.33
Thalamus	0.706	0.313–1.682	0.413
Lobe	0.986	0.434–2.36	0.973
Hematoma volume	1.037	1.026–1.049	<0.001
IVE	0.849	0.58–1.239	0.397
**Laboratory testing**
WBC	1.020	0.967–1.074	0.466
IL-6	1.002	0.992–1.013	0.639
PCT	0.701	0.427–0.986	0.099
Hcy	1.129	1.099–1.161	<0.001
CRP	0.973	0.948–0.997	0.034
Surgery	1.432	0.946–2.15	0.086
Intubation	2.881	1.825–4.534	<0.001
**Multivariate logistics**	
**regression (Stepwise)**
Age	–	–	–
Diabetes mellitus	–	–	–
GCS score	–	–	–
CRP	–	–	–
Intubation	3.479	2.062–5.887	<0.001
Hcy	1.122	1.091–1.156	<0.001
Hematoma volume	1.031	1.018–1.043	<0.001

*Univariate analysis revealed significant differences in the history of diabetes mellitus (p = 0.011), age (p = 0.04), baseline GCS scores (p < 0.001), baseline hematoma volume (p < 0.001), intubation (p < 0.001), levels of CRP (p = 0.034), and Hcy (p < 0.001) for HAP prediction. Subsequently, the multivariate logistic regression analysis showed that Hcy [odds ratio (OR): 1.122, 95% confidence interval (CI): 1.091–1.156, p < 0.001], intubation (OR: 3.479, 95% CI: 2.062–5.887, p < 0.001), and hematoma volume (OR: 1.031, 95% CI: 1.018–1.043, p < 0.001) were associated with HAP. Of note, there was still an independent association between Hcy and HAP after the factors of intubation and hematoma volume adjusted*.

### Hcy as a Predictor for Poor Outcome

A total of 173 (29.9%) patients had poor outcomes after a 3-month follow-up. Patients with ICH having poor outcomes had much higher Hcy compared with those with good outcomes (*p* < 0.001; Figure 2B). Univariate analysis revealed significant differences in age (*p* = 0.032), baseline GCS scores (*p* < 0.001), baseline hematoma volume (*p* < 0.001), Hcy (*p* < 0.001), and HAP (*p* < 0.001) for predicting poor outcomes. Subsequently, the predictors (candidates for independent variables based on the results of univariate regression analysis: age, baseline GCS scores, baseline hematoma volume, Hcy, and HAP) were included in a stepwise multiple logistic regression. The results showed that Hcy (OR: 1.072, 95% CI: 1.044–1.101, *p* < 0.001), HAP (OR: 1.638, 95% CI: 1.025–2.599, *p* = 0.037), and hematoma volume (OR: 1.038, 95% CI: 1.026–1.050, *p* < 0.001) were associated with poor outcomes. Of note, there was still an independent association between Hcy and poor outcomes after the factors of HAP and hematoma volume adjusted (Table 3). The ROC was generated to examine the ability of Hcy to predict the poor functional outcomes at 3 months, with an AUC of 0.689 with 95% CI: 0.639–0.739 (*p* < 0.001; Figure 3B). Moreover, the optimal cutoff value of Hcy for predicting poor outcomes was 19.45 μmol/l, which yield a sensitivity of 66.5%, a specificity of 67.0%, PPV of 46.2%, NPV of 82.4%, and accuracy of 66.8% (Table 4).

**Table 3:** Univariate and multivariate analyses of the potential predictors for poor outcomes.

**Univariate selection**	**3-Month poor outcomes**		
	**OR**	**95% CI**	***p***
Age	1.017	1.002–1.034	0.032
Sex	0.844	0.566–1.245	0.397
**Medical history**
Hypertension	0.961	0.655–1.422	0.841
Coronary heart disease	1.240	0.786–1.929	0.346
Hyperlipidemia	1.156	0.792–1.679	0.448
Diabetes mellitus	0.879	0.584–1.308	0.529
Smoking	0.800	0.549–1.171	0.247
Alcoholism	0.805	0.562–1.15	0.234
**Clinical status on admission**
Blood sugar	1.051	0.994–1.11	0.078
SBP	1.001	0.995–1.007	0.787
DBP	0.999	0.988–1.009	0.796
GCS score	0.829	0.758–0.906	<0.001
NIHSS score	0.994	0.971–1.017	0.619
**Radiographic status on admission**
**Hematoma location**
Cerebellum	0.438	0.121–1.411	0.18
Basal ganglia	0.728	0.345–1.596	0.412
Thalamus	0.782	0.359–1.763	0.541
Lobe	0.750	0.337–1.722	0.486
Hematoma volume	1.045	1.034–1.058	<0.001
IVE	0.820	0.572–1.172	0.277
**Laboratory testing**
WBC	0.972	0.923–1.023	0.281
IL-6	1.000	0.99–1.01	0.999
PCT	0.683	0.431–0.951	0.062
Hcy	1.095	1.07–1.123	<0.001
CRP	0.984	0.961–1.006	0.158
Surgery	1.389	0.937–2.049	0.099
HAP	3.588	2.417–5.345	<0.001
Intubation	1.085	0.673–1.719	0.732
**Multivariate logistics regression (stepwise)**	
Age	–	–	–
GCS score	–	–	–
Hcy	1.072	1.044–1.101	<0.001
HAP	1.638	1.025–2.599	0.037
Hematoma volume	1.038	1.026–1.050	<0.001

*Univariate analysis revealed significant differences in age (p = 0.032), baseline GCS scores (p < 0.001), baseline hematoma volume (p < 0.001), Hcy (p < 0.001), and HAP (p < 0.001) for predicting poor outcomes. And multivariate logistic regression analysis demonstrated that Hcy was independently associated 3-month poor outcome (OR: 1.082, 95% CI: 1.056–1.110, p < 0.001) in ICH after the factors of HAP and hematoma volume adjusted*.

**Table 4:** The cut-off points and accuracy of Hcy to predict HAP and poor outcome.

	**Cut-Off point**	**Sensitivity (%)**	**Specificity (%)**	**PPV (%)**	**NPV (%)**	**Accuracy (%)**
HAP	18.60	81.3	64.6	43.2	91.2	68.7
Poor outcome	19.45	66.5	67.0	46.2	82.4	66.8

*The cut-off point for predicting HAP was 18.60 μmol/l with the sensitivity, specificity, PPV, NPV, and accuracy of 81.3, 64.6, 43.2, 91.2, and 68.7%, respectively; the optimal cutoff value of Hcy for predicting poor outcome was 19.45 μmol/l, which yield a sensitivity of 66.5%, a specificity of 67.0%, PPV of 46.2%, NPV of 82.4%, and accuracy of 66.8%*.

### Linear Correlation Between Hcy and the Risk of HAP as Well as Poor Outcome

**Figure 4:**
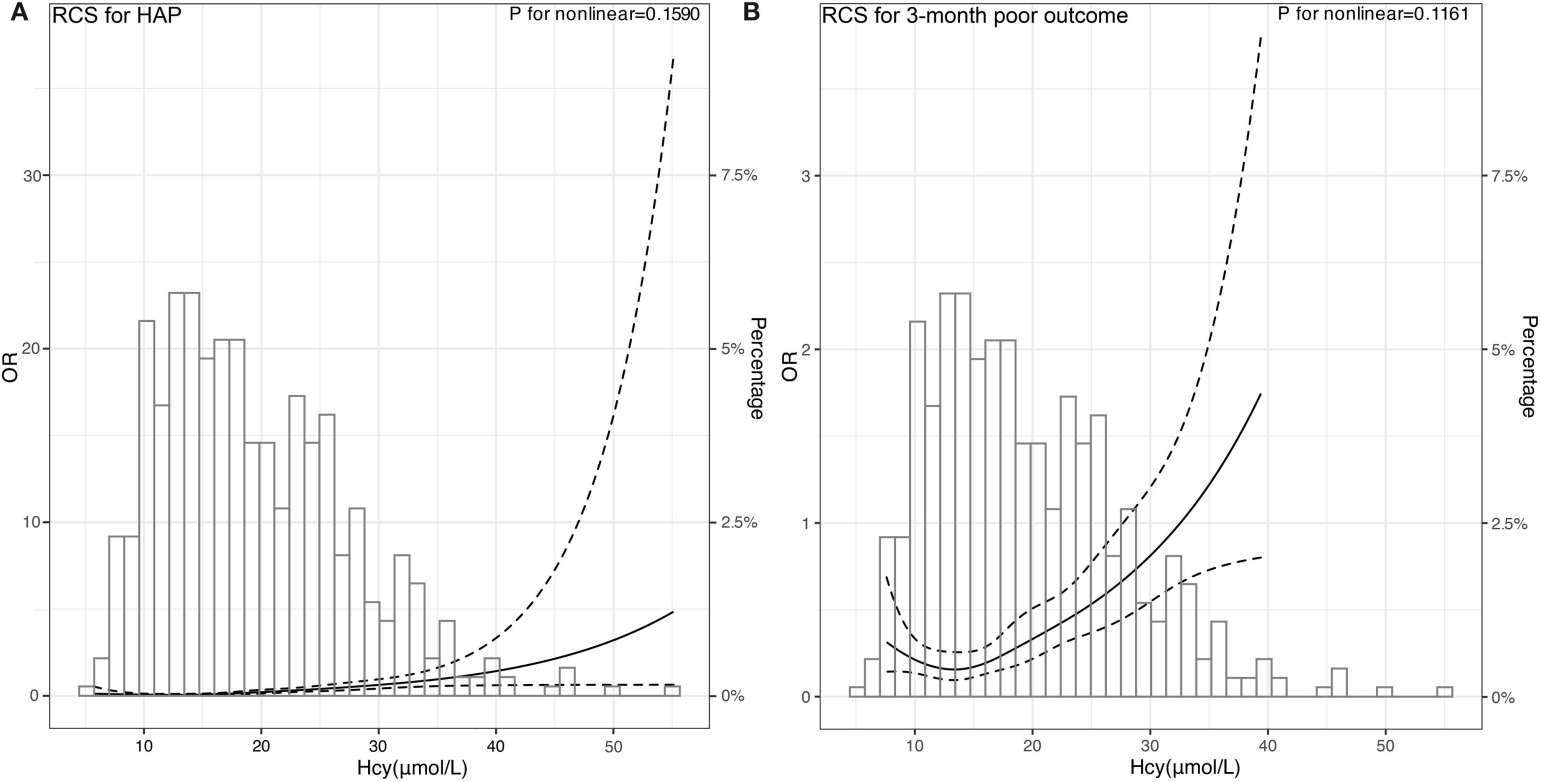
Association of Hcy with risk of HAP and poor 3-month poor outcome. OR and 95% CI were derived from restricted cubic spline regression, with knots placed at 5th, 27.5th, 50th, 72.5th, and 95th percentiles of Hcy. The solid line represents the OR and the dashed lines represent the 95% CI. **(A)** OR of RCS for HAP was adjusted for the age, sex, and variables selected by stepwise logistics regression including intubation and hematoma volume. **(B)** OR of RCS for 3-month poor outcome was adjusted for the age, sex, and variables selected by stepwise logistics regression including HAP and hematoma volume.

Subsequently, we used RCSs to flexibly model and visualize the relationship between Hcy and HAP and poor outcomes. With the increase of Hcy levels, the risk of HAP and poor outcomes increased gradually. When the Hcy was at the highest level, the risk of HAP and poor outcomes was the highest. The figure showed an ascending trend of Hcy levels with a risk of HAP and poor outcome [respective P for non-linearity = 0.1590 (Figure 4A) and 0.1161 (Figure 4B)].

In addition, we found that the mediating effect of HAP on the relationship between Hcy and poor outcomes was not significant (Table 5).

**Table 5:** The mediation of the effect of Hcy on the poor outcome by HAP.

	**Estimate**	**95% CI**	***p*** **-value**
Direct effect	0.0057	(0.0045–0.0100)	<0.001
Indirect effect	0.0003	(−0.0015–0.0000)	0.770
Total effect	0.0060	(0.0038–0.0000)	<0.001
Prop. mediated	0.0566	(−0.3662–0.3200)	0.770

*The mediating effect of HAP on the relationship between Hcy and poor outcomes was not significant (p = 0.770)*.

## Discussion

In this study, we found that high plasma Hcy was independently associated with HAP and poor outcomes in patients with primary ICH. The risk for HAP and poor outcomes increased with higher Hcy levels. Furtherly, ROC analysis indicated good associations between high homocysteine levels and the development of HAP and poor outcomes. However, the association between Hcy and poor outcomes was not mediated by HAP.

Homocysteine (Hcy) is a sulfur-containing amino acid related to methionine metabolism. There are several studies on associations between Hcy and inflammation. Two studies from China found that elevated serum levels of CRP and Hcy were associated with the risk of developing post-stroke depression (6, 14). Elevated Hcy was a marker of spontaneous bacterial peritonitis in patients with cirrhotic ascites (15). You et al. found that a higher level of homocysteine at admission was independently associated with in-hospital pneumonia, in-hospital mortality, and poor functional outcome in the hospital (10). Likewise, we demonstrated that Hcy was independently associated with HAP in ICH. The results showed that the patients with elevated Hcy levels had a 1.12-fold increased risk of HAP after ICH.

Meanwhile, our findings indicated that Hcy was independently associated with a 3-month poor outcome in ICH. And we found elevated Hcy level was associated with a 1.08-fold increased risk of poor functional outcomes. To date, the results from several studies on the association between them were contradictory. High Hcy level was independently associated with a poorer 3-month prognosis and a lower survival rate within 1 year in patients with ICH (16), which was partly consistent with our study. However, a study including 69 participants with ICH indicated that Hcy levels might not be predictors of the 6-month clinical outcome in patients (17). Variations in sample size, population, the definition of clinical outcomes, and different statistic methods may have led to discrepant findings from these studies. Of note, the association between Hcy and poor outcomes was not mediated by HAP.

Furtherly, the predictive accuracy of Hcy for HAP was evaluated. Our results showed that Hcy had moderate predictive accuracy in HAP and poor outcome through ROC analysis. Moreover, we determined that the best cut-off value of Hcy to predict HAP and poor outcomes were, respectively, 18.60 and 19.45 μmol/l. Based on this finding, we suggest that more attention should be paid to the development of HAP when the Hcy level is higher than 18.60 μmol/l. In addition, our data showed that the risk of HAP and poor outcome increased gradually with the increase of Hcy levels, revealing a linear relationship between them. This easily-acquired marker can help clinical workers recognize early the occurrence of HAP after ICH. The mechanisms may be that Hcy is involved in the induction of inflammatory determinants, namely, the expression of adhesion molecules, leukocyte adhesion, endothelial dysfunction, oxidative stress, and reduced nitric oxide bioavailability (18–22). Nevertheless, the exact mechanisms underlying the relationship between elevated Hcy levels and HAP and poor functional outcome after ICH are not yet fully understood, which warrant further studies.

Our work first investigated the association between Hcy and HAP in patients with primary ICH and demonstrates that Hcy is independently associated with HAP and poor outcomes. Distinct from the markers aforementioned, Hcy is a widely available and inexpensive biomarker that may be used to stratify the risk of HAP in clinical practice. Also, Hcy may be a potential candidate for a new prediction score combined with other predictors to increase sensitivity and specificity. This easily-acquired marker can help clinical workers recognize early the development of HAP and poor outcome after ICH. Therefore, the prospective studies investigating the role of homocysteine reduction in ICH may be justified.

The limitations of this study should be concerned. First, the patients were all from the south of China, whose similar dietary habits may influence the level of Hcy. So some bias may exist because of the population. Second, the sample size was relatively small and only Chinese patients were evaluated, which may limit the generalizability of the study results in other cohorts. Therefore, further study of global multicenter with a large sample size should be performed to avoid the above limitations. Third, we did not collect the levels of plasma vitamin B and folate among patients and the therapeutic method of Hcy during the follow-up. Finally, the variables such as duration of intubation and length of hospital/ICU stay which is relevant for the outcomes were not included in this study. To the best of our knowledge, this is the first study to investigate the association between Hcy and HAP in patients with primary ICH.

## Conclusion

In conclusion, our study suggests that an elevated Hcy level is independently associated with both HAP and 3-month poor functional outcomes after primary ICH. And there are linear relationships between Hcy and HAP and poor outcomes, which show an increased risk of HAP and poor outcomes with the elevation of Hcy.

## Data Availability Statement

The raw data supporting the conclusions of this article will be made available by the authors, without undue reservation.

## Ethics Statement

The studies involving human participants were reviewed and approved by the Institutional Ethical Committee of First Affiliated Hospital of University of South China. The patients/participants provided their written informed consent to participate in this study.

## Author Contributions

JP: conception, design, drafting of manuscript, and final approval of manuscript. GZ and SX: acquisition of data, design, drafting of manuscript, and final approval of manuscript. SL: design, analysis and interpretation of data, drafting of the manuscript, revision of manuscript, and final approval of manuscript. All authors contributed to the article and approved the submitted version.

## Funding

This work was partly supported by Hunan Provincial Science and Technology Department (2021JJ40477).

## Conflict of Interest

The authors declare that the research was conducted in the absence of any commercial or financial relationships that could be construed as a potential conflict of interest.

## Publisher's Note

All claims expressed in this article are solely those of the authors and do not necessarily represent those of their affiliated organizations, or those of the publisher, the editors and the reviewers. Any product that may be evaluated in this article, or claim that may be made by its manufacturer, is not guaranteed or endorsed by the publisher.

## Abbreviations

ICH: intracerebral hemorrhage
HAP: hospital-acquired pneumonia
Hcy: homocysteine
M: mean
GCS: Glasgow coma scale
IQR: interquartile range
CRP: C-reaction protein
ROC: receiver operating characteristic
RCS: restricted cubic spline
AUC: area under the ROC curve.

## References

[1] Cordonnier C Demchuk A Ziai W Anderson CS. Intracerebral haemorrhage: current approaches to acute management. Lancet. (2018) 392:1257–68. 10.1016/S0140-6736(18)31878-6 30319113

[2] Alsumrain M Melillo N Debari VA Kirmani J Moussavi M Doraiswamy V. Predictors and outcomes of pneumonia in patients with spontaneous intracerebral hemorrhage. J Intensive Care Med. (2013) 28:118–23. 10.1177/0885066612437512 22337709

[3] Lord AS Langefeld CD Sekar P Moomaw CJ Badjatia N Vashkevich A. Infection after intracerebral hemorrhage: risk factors and association with outcomes in the ethnic/racial variations of intracerebral hemorrhage study. Stroke. (2014) 45:3535–42. 10.1161/STROKEAHA.114.006435 25316275 PMC4245453

[4] Morotti A Marini S Jessel MJ Schwab K Kourkoulis C Ayres AM. Lymphopenia, infectious complications, and outcome in spontaneous intracerebral hemorrhage. Neurocrit Care. (2017) 26:160–6. 10.1007/s12028-016-0367-2 28004330 PMC5336513

[5] Marini S Morotti A Lena UK Goldstein JN Greenberg SM Rosand J. Men experience higher risk of pneumonia and death after intracerebral hemorrhage. Neurocrit Care. (2018) 28:77–82. 10.1007/s12028-017-0431-6 28730561 PMC5775939

[6] Cheng LS Tu WJ Shen Y Zhang LJ Ji K. Combination of high-sensitivity c-reactive protein and homocysteine predicts the post-stroke depression in patients with ischemic stroke. Mol Neurobiol. (2018) 55:2952–8. 10.1007/s12035-017-0549-8 28456936

[7] Elsherbiny NM Sharma I Kira D Alhusban S Samra YA Jadeja R. Homocysteine induces inflammation in retina and brain. Biomolecules. (2020) 10:393. 10.3390/biom10030393 32138265 PMC7175372

[8] Li JJ Li Q Du HP Wang YL You SJ Wang F. Homocysteine triggers inflammatory responses in macrophages through inhibiting CSE-H2S signaling via DNA hypermethylation of CSE promoter. Int J Mol Sci. (2015) 16:12560–77. 10.3390/ijms160612560 26047341 PMC4490461

[9] Kumar M Sandhir R. Hydrogen sulfide suppresses homocysteine-induced glial activation and inflammatory response. Nitric Oxide. (2019) 90:15–28. 10.1016/j.niox.2019.05.008 31146011

[10] You S Wang L Du H Zheng D Zhong C Wu Q. Elevated total homocysteine predicts in-hospital pneumonia and poor functional outcomes in acute ischemic stroke. Curr Neurovasc Res. (2020) 17:745–53. 10.2174/1567202617666201214111244 33319686

[11] Hanley DF Thompson RE Rosenblum M Yenokyan G Lane K McBee N. Efficacy and safety of minimally invasive surgery with thrombolysis in intracerebral haemorrhage evacuation (MISTIE III): a randomised, controlled, open-label, blinded endpoint phase 3 trial. Lancet. (2019) 393:1021–32. 10.1016/S0140-6736(19)30195-3 30739747 PMC6894906

[12] Lanks CW Musani AI Hsia DW. Community-acquired pneumonia and hospital-acquired pneumonia. Med Clin North Am. (2019) 103:487–501. 10.1016/j.mcna.2018.12.008 30955516

[13] Guerci P Bellut H Mokhtari M Gaudefroy J Mongardon N Charpentier C. Outcomes of *Stenotrophomonas maltophilia* hospital-acquired pneumonia in intensive care unit: a nationwide retrospective study. Crit Care. (2019) 23:371. 10.1186/s13054-019-2649-5 31752976 PMC6873544

[14] Yin J Zhong C Zhu Z Bu X Xu T Guo L. Elevated circulating homocysteine and high-sensitivity C-reactive protein jointly predicts post-stroke depression among Chinese patients with acute ischemic stroke. Clin Chim Acta. (2018) 479:132–7. 10.1016/j.cca.2018.01.011 29325799

[15] Abdel-Razik A Eldars W Elhelaly R Eldeeb AA Abdelsalam M El-Wakeel N. Homocysteine: a new diagnostic marker in spontaneous bacterial peritonitis. Eur J Gastroenterol Hepatol. (2018) 30:779–85. 10.1097/MEG.0000000000001109 29505476

[16] Wang D Wang W Wang A Zhao X. Association of severity and prognosis with elevated homocysteine levels in patients with intracerebral hemorrhage. Front Neurol. (2020) 11:571585. 10.3389/fneur.2020.571585 33193018 PMC7604273

[17] Zhou F Chen B Chen C Huang J Chen S Guo F. Elevated homocysteine levels contribute to larger hematoma volume in patients with intracerebral hemorrhage. J Stroke Cerebrovasc Dis. (2015) 24:784–8. 10.1016/j.jstrokecerebrovasdis.2014.11.005 25620712

[18] Albu E Filip C Zamosteanu N Jaba IM Linic IS Sosa I. Hyperhomocysteinemia is an indicator of oxidant stress. Med Hypotheses. (2012) 78:554–5. 10.1016/j.mehy.2012.01.007 22285627

[19] Stühlinger MC Tsao PS Her JH Kimoto M Balint RF Cooke JP. Homocysteine impairs the nitric oxide synthase pathway: role of asymmetric dimethylarginine. Circulation. (2001) 104:2569–75. 10.1161/hc4601.098514 11714652

[20] Chang PY Lu SC Lee CM Chen YJ Dugan TA Huang WH. Homocysteine inhibits arterial endothelial cell growth through transcriptional downregulation of fibroblast growth factor-2 involving G protein and DNA methylation. Circ Res. (2008) 102:933–41. 10.1161/CIRCRESAHA.108.171082 18309099

[21] Currò M Gugliandolo A Gangemi C Risitano R Ientile R Caccamo D. Toxic effects of mildly elevated homocysteine concentrations in neuronal-like cells. Neurochem Res. (2014) 39:1485–95. 10.1007/s11064-014-1338-7 24867323

[22] Sharma M Tiwari M Tiwari RK. Hyperhomocysteinemia: impact on neurodegenerative diseases. Basic Clin Pharmacol Toxicol. (2015) 117:287–96. 10.1111/bcpt.12424 26036286

